# Research on the Influence of Interface Visual Design Features of Mobile News on Cognitive Load: A Study of Elderly Users in China

**DOI:** 10.3390/bs16010032

**Published:** 2025-12-23

**Authors:** Chang Liu, Qing-Xing Qu

**Affiliations:** 1 School of Economics and Management, Shenyang Aerospace University, Shenyang 110136, China; mynamell@163.com; 2 Department of Industrial Engineering, School of Business Administration, Northeastern University, Shenyang 110167, China

**Keywords:** elders, mobile news interface, visual design, cognitive load, eye movement behavior

## Abstract

This study addresses specific gaps in current research on user-experience interface design for news and information apps targeted at elderly users, particularly in the context of human factors and ergonomics. To investigate how interface design features of mobile news clients affect the cognitive load of elderly users, an in-depth analysis was conducted using a combination of objective eye movement tests and subjective evaluation scales. Mobile news client interfaces with systematically varied visual complexity were designed by orthogonally manipulating three core elements identified from top-ranked Chinese news apps and prior literature, and within-subject repeated experiments were performed to collect subjective cognitive load data, objective eye movement data, and behavioral data, validating the proposed hypothesis model. The results indicate that the visual complexity of mobile news client interfaces significantly impacts the cognitive load of elderly users, with keyword color substantially modulating this effect. These findings contribute to the knowledge base on mobile news client interface design for elderly users and provide practical recommendations for designers to create more equitable interfaces, enhancing usability for this demographic.

## 1. Introduction

According to the 52nd Statistical Report on the Development of the Internet in China released by the China Internet Network Information Center (CNNIC), as of December 2023, the proportion of Chinese netizens using mobile phones to access the Internet was 99.8%, while the proportion of netizens aged 60 and above was 14.3%, and the Internet is further penetrating into the elderly group (CNNIC, 2023). Compared with the complexity of computer operation, smart phone operation is simple and convenient, and the elderly can easily use smart phones for online shopping, watching news, watching short videos and other leisure and entertainment activities, among which “news information” is one of the most favorite mobile phone applications of the elderly. The data show that the elderly use news apps for more than 15% of the total use time of all apps, which is 1.8 times the average use time of all netizens (Q. Z. Wang et al., 2020). However, due to certain differences between the physiological and cognitive characteristics of the elderly and the middle-aged and young people, the use of new media will bring cognitive and operational obstacles to the elderly users. How to take into account the characteristics of different groups to make the interface design of the news client more inclusive to reduce the cognitive load of the elderly in the operation process, and to effectively improve the experience of elderly users when using mobile applications are urgent problems to be solved by application operators.

Most of the design only focuses on font size, brightness adjustment, etc., which is only one of the factors that lead to poor experience for the elderly, and does not take into account the unique physiological and cognitive characteristics of the elderly, which determines their weak cognitive ability (Chen, 2021; C. Liu et al., 2018; Zhu et al., 2023). The interface of mobile news apps is generally filled with symbols, text, pictures, and other information. A high cognitive load for the elderly during use might result from too complicated interfaces, too much information, complex information interactions, and layouts that do not match their cognitive patterns, therefore influencing their experience (Chen, 2021). In daily life, elderly users of mobile news apps often use them before bed, while waiting for buses or during commutes. Their main goal is to find information they are curious in; hence, visual search is quite important. The efficiency of their searches directly impacts the efficiency of information acquisition, further influencing the cognitive load and user experience when using these products (Zhu et al., 2023). Elderly people are a special group, where visual, memory, reasoning, and other abilities will decline with age (C. Liu et al., 2018). Therefore, the study on the visual design of the interface of mobile news client for the elderly is particularly crucial.

Studies by certain academics have revealed that using mobile phones caused eye fatigue more readily than using computers (Jaiswal et al., 2019). On one hand, the closer distance between eyes and mobile phone screens when using mobile phones was more likely to induce visual fatigue. On the other hand, the interface design itself also affects users’ visual perception, in which the visual complexity of the interface design played a very important role in impacting users’ visual perception (X. S. Wang & Li, 2023). The whole interface design of the mobile news clients was the surrounding environment relevant to the target information that users look for, and visual search was largely influenced by the environmental complexity. For elderly mobile phone users with declining visual abilities, this undoubtedly had a greater impact than on younger people. Nevertheless, at this stage, most of the study population and products emphasizing visual complexity were aimed toward young people and computer-based website interfaces. Since the number of studies on the visual complexity of mobile interface design and the elderly group was still quite limited (W. Liu et al., 2022; King et al., 2020; Tong et al., 2022), it is highly crucial to investigate the visual difficulty of the interface design of mobile news client for the elderly users.

Furthermore, three primary categories of cognitive load evaluation methods exist: task performance measurement, subjective assessments, and physiological measurements. Pass contended that cognitive load was multidimensional, thus it was more reasonable to apply multiple techniques to completely evaluate cognitive load than depending just on a single method or index (Vanneste et al., 2021). Meanwhile, based on the consensus of views of domestic and foreign scholars on cognitive load measurement methods, it was believed that the use of subjective–objective combination of measurement methods can avoid the bias of the results due to the error of a certain measurement method (Zheng et al., 2024). For example, Zheng et al. (2024) combined two types of physiological measurement techniques, namely, eye movement and electrooculogram, with subjective and performance measures to objectively discriminate and predict the mental load of the workers over time to reduce the rate of accidents and enhance production efficiency; Huang et al. (2024) examined drivers’ mental workload in a simulated auditory-based dual-task driving scenario, and the multimodal physiological signals, including electroencephalography (EEG), electrocardiography (ECG), and electrodermal activity (EDA), as well as the subjective measurements, were used as a comprehensive index. The results showed that the composite index was more suitable for measuring the change in cognitive load size than a single index; Shrisowmya et al. (2019) used a classification model to link the physiological indicators and subjective load data and combined them into a composite assessment index, and found that the composite index could more accurately assess the level of cognitive load of the subjects under different workloads than a single index.

This study is theoretically grounded in an integrated framework combining Cognitive Load Theory (CLT) from human–computer interaction with visual perception and cognitive aging perspectives. CLT provides the foundational premise that interface designs inducing high extraneous cognitive load may exceed working memory capacities—particularly among elderly users experiencing age-related declines in visual acuity, contrast sensitivity, and attentional control (Ran et al., 2021; Vanneste et al., 2021). Complementing this, visual perception theories underscore how design elements such as color contrast and layout complexity affect information processing efficiency, with empirical evidence suggesting that reduced perceptual clutter facilitates faster target identification in older adults (Huang et al., 2024). Further informed by cognitive aging theories, which document sensory and memory degradation in older populations, we argue that smartphone interfaces must minimize cognitive demands to promote usability and reduce fatigue (Shrisowmya et al., 2019). Synthesizing these perspectives, this research investigates how visual complexity and keyword color in mobile news apps influence cognitive load, thereby connecting theoretical mechanisms to observable search behaviors and performance metrics in elderly users.

On the whole, the current research results provide a good theoretical basis and practical value, but there are also some defects: (1) there are few studies on the design of mobile client news interface from the perspective of the elderly; (2) there are few studies on the cognitive load of the elderly in the process of using mobile client interface from the perspective of visual search; and (3) there are few evaluation methods specifically for the cognitive load of the elderly mobile terminal interface. In view of the above shortcomings, this study will identify mobile client interface design schemes with different design elements based on the relevant theories of web interface design, and comprehensively use the self-report method, eye-tracking technology, and the behavioral expression method to evaluate the cognitive load of mobile news client interfaces for elderly users. Therefore, the relationship between different interface design features of mobile news clients and their influence is investigated. In the absence of research on how specific design features of mobile client interface affect cognitive load of elderly users at home and abroad, the research in this paper will enrich the research on mobile client interface design for elderly users, and also have reference significance for how mobile interface designers can rationally match design elements to better attract elderly users and effectively achieve business purposes.

## 2. Research Hypothesis

In less than 50 milliseconds, users have an initial “gut feeling” when using a website that helps them decide whether to stay or leave, and this first impression can depend on many elements, with the visual complexity of the interface design largely determining the user’s “gut feeling”. The interface design’s visual complexity mostly controls this “intuition” of the users (Collaud et al., 2022). Therefore, from the point of view of academic research progress, there are many research results on the complexity of interface design, yet, at this stage, the number of research on the visual complexity of mobile product interfaces is still very limited. For example, Tong et al. (2022) studied the relationships between live background visual complexity, emotional states, and purchase intention. The results indicated that the background visual complexity of the livestream room influences consumers’ purchase intention positively via the evoked emotional states (pleasure and arousal); W. Liu et al. (2022) discussed the factors affecting webpage complexity on users’ emotional experience from the three aspects of cognition, content, and form; King et al. (2020) studied how web design visual complexity—feature complexity and design complexity—influenced the perceptions of visual informativeness, cues for engagement, favorable initial impressions, and behavioral intentions; Q. Z. Wang et al. (2020) investigated the influence of the background complexity of a product image on consumer attention, information processing, and purchase intention using eye-tracking and questionnaire methods, and the results showed that product images with higher background complexity attract greater attention. While some academics claimed that very complex web pages increased the depth of information given and had higher user satisfaction, therefore positively altering users’ online activity, others stated that simple web pages have greater ease of use and aesthetics (King et al., 2020; Ramezani Nia & Shokouhyar, 2020). In this study, we will design mobile news client interfaces with different visual complexity obtained by combining different elements in order to investigate the effect of mobile news client interface design features on the cognitive load of elderly users and to propose research hypothesis H1.

**H1.**  *The visual complexity of mobile news client interface design has a significant effect on the cognitive load of elderly users.*

In addition, it has been found that font color played a very important role in influencing visual search behavior, reading effect, learning efficiency, cognitive resource allocation, etc. (Kuo et al., 2022; Wallace et al., 2022; Zhou et al., 2022). Usually, fonts with color will attract users’ attention more easily, reduce unnecessary visual searches, and enable readers to capture the text focus more quickly. In addition to its role in vision, font color emphasis also affects many aspects of readers’ language comprehension, helping readers construct discourse topic structure representations in reading. For example, in Yu et al.’s (2018) eye-movement study on browsing multimedia with different fonts and font color combinations, it was found that there were significant differences in the eye movement indexes of subjects browsing different colors and fonts. In terms of reading time, blue bold fonts were the shortest and easiest to browse, and black bold fonts were the longest and most difficult to browse. In terms of the gaze time, blue bold fonts and red bold fonts were significantly more intelligible than other types of emphasis. It was finally concluded that a reasonable combination of different fonts and colors could increase the readability of multimedia texts, thus improving learners’ learning efficiency. Wu et al. (2021) in their study on how visual salience affected salient information processing found that, at the vocabulary level, font color emphasis promoted vocabulary recognition and subsequent semantic integration, resulting in finer semantic processing of vocabulary. Wu et al. (2023) in their study on the effect of font color emphasis on the sentence comprehension of the elderly found that font color emphasis reduced the allocation of resources to the elderly at the lexical level. In a comprehensive analysis of the above, font color affected the user’s interface visual behavior, cognitive resource allocation, and vocabulary processing and understanding, which in turn affected the user’s attitude of eventual use. In visual processing, vocabulary recognition, language comprehension and other abilities, the elderly show signs of partial weakening of the text of the easy to read and have higher readability requirements compared to young people (Iancu & Iancu, 2020). Therefore, it is necessary to do further studies on the regulating effect of font color on the influence of mobile news client interface design complexity on cognitive load of elderly users, and propose the research hypothesis H2.

**H2.**  *Font color moderates the effect of mobile news client interface design visual complexity on the cognitive load of elderly users.*

The selection of red and black as the font color variables in this study is grounded in visual perception and cognitive aging research, which highlights the importance of high-contrast emphasis for elderly users facing age-related declines in color discrimination, particularly in the blue spectrum due to lens yellowing that filters blue light, making blues appear darker and less distinguishable from dark colors like black or navy (Kuo et al., 2022; Wallace et al., 2022; Zhou et al., 2022). Black was chosen as the default font color, representing standard low-contrast text that may increase perceptual effort and cognitive load for seniors with reduced contrast sensitivity (Yu et al., 2018). Red, conversely, was selected for its high visibility and alerting properties, providing strong contrast on light backgrounds to facilitate faster target identification and reduce search times, as red maintains better discriminability in aging vision compared to blues, which are often recommended in general readability studies (Wu et al., 2021) but may exacerbate visibility issues for elderly due to diminished blue–green sensitivity (Iancu & Iancu, 2020; Wu et al., 2023). Other colors like blue were excluded to focus on a controlled comparison of default (black) versus emphasis (red), common in mobile interfaces for highlights, while avoiding colors prone to age-specific perceptual degradation; future research could incorporate blue for direct comparisons to validate these effects.

**Figure 1:**
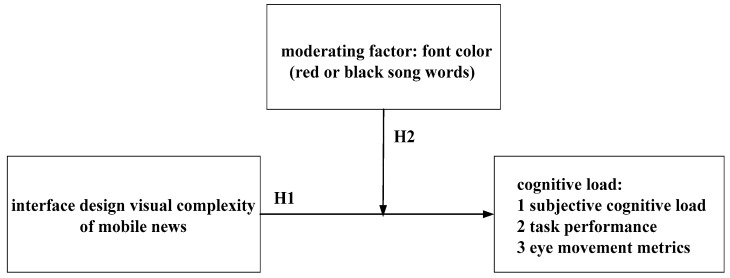
The influence model of mobile news interface design features on cognitive load of elderly users.

In summary, this study aims to investigate the effects of visual complexity in different mobile news client interface designs on the cognitive load of elderly users, as well as the regulation mechanism of font color, using eye-tracking technology. Based on this, the following research hypotheses and conceptual model are proposed, as illustrated in Figure 1.

## 3. Methodology

### 3.1. Participants

Participants were recruited via convenience sampling from community centers in urban China, ensuring familiarity with mobile news apps (all had used such apps at least once in the past three months, with an average weekly usage of 2–3 h). Inclusion criteria required normal or corrected-to-normal vision (e.g., via glasses or contact lenses, verified through self-report and a pre-experiment visual acuity test), no history of neurological disorders, and basic literacy (education level: 58% high school or equivalent, 42% college or higher). In this study, “older adults” were defined as individuals aged 55 and above. Although many Western ergonomics and gerontological studies use 60 or 65 as the lower boundary, a growing body of human-factors research focused on technology adoption and interface design in China and other East Asian countries adopts 55+ as the threshold because (a) China’s official retirement age is 55 for women and 60 for men, and many individuals experience functional aging and begin intensive smartphone use immediately after retirement; (b) national policy and industry guidelines for “age-appropriate” digital products explicitly target citizens aged 55 and older; and (c) recent empirical studies of mobile app usability and cognitive load in Chinese samples consistently use 55+ to capture the population that most actively migrates to smartphone-based news consumption post-retirement (C. Liu et al., 2018; Zhu et al., 2023; Chen, 2021). This cutoff therefore better reflects the sociocultural and functional aging realities in the study’s context while still encompassing the age range (60+) typically associated with pronounced declines in contrast sensitivity, working memory, and selective attention.

The sample size of 24 (12 males, 12 females, mean age 61.9 ± 4.8 years) was determined a priori using G*Power software (version 3.1.9.7) for repeated-measures ANOVA (F-test), assuming a medium effect size (f = 0.25), α = 0.05, and power = 0.80, yielding a minimum of 11 participants (Cohen, 1988; Faul et al., 2007). Our sample exceeds this, aligning with typical sizes in eye-tracking studies on elderly cognitive load, which prioritize intensive data collection over large-scale representation. To ensure ANOVA assumptions, we tested for normality using Shapiro–Wilk tests (all *p* > 0.05 for subjective cognitive load, pupil diameter, fixation count, blink frequency, and task completion time) and homogeneity of variance using Mauchly’s test of sphericity (*p* > 0.05 for all measures; Greenhouse–Geisser corrections applied where needed). While not intended to represent China’s entire 1.4 billion population or 200+ million elderly, this controlled experimental sample suffices for detecting within-subject effects in visual perception and cognitive load, with generalizability limited to urban, literate elderly app users; future studies should employ stratified sampling for broader representation. Ethical approval was obtained from the Institutional Review Board, with all participants providing written informed consent after being informed of study procedures, risks, and rights.

### 3.2. Selection of Experimental Materials

In traditional research on website complexity, numerous factors affected visual complexity, such as the amount of information presented on the webpage interface, including text, images, animations, and the arrangement of these elements, also known as the layout of the webpage interface (Guo et al., 2022; Zhang et al., 2024). This study first selected the top ten news and information application (APP) brands based on the comprehensive ranking of the China National Product Promotion (CNPP) Brand List as the initial materials for experimental webpage interfaces. Then, an expert focus group was formed to observe the selected news client interfaces and draw on previous research literature on visual complexity of webpage interfaces to determine the main design elements and their levels for the experimental mobile news client interface visual complexity design. The design elements were combined to create the experimental webpage interfaces, as detailed in Table 1 and Table 2. To enhance the representativeness of each interface in terms of design elements and their levels, Mockplus (version 3.7.2.0), a design software, was used to create prototypes of the applications. The content of the navigation bar and function bar was controlled, and only the main design elements differed in their levels. Ultimately, 16 mobile news client interfaces with different visual complexities were designed, each containing 12 identical news items. To eliminate memory effects, the position of all target news items was the same, while the positions of non-target news items were different. The news client interfaces were downloaded to experimental smartphones in offline file format.

**Figure 2:**
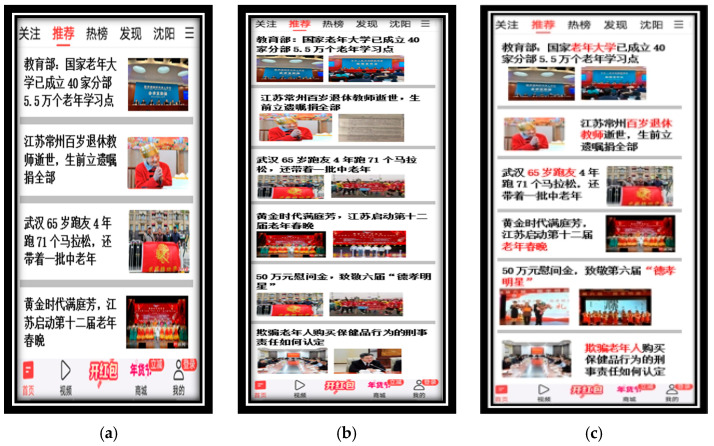
Examples of experimental mobile news client interfaces illustrating the three levels of visual complexity. (**a**) Low visual complexity—Interface 1 (3–4 news items per screen, left-text-right-image layout); (**b**) medium visual complexity—Interface 5 (5–6 news items per screen, upper-text-lower-image layout); (**c**) high visual complexity—Interface 9 (7–8 news items per screen, hybrid/mixed layout). All interfaces are from the black-keyword conditions (no red emphasis on keywords) to isolate the effects of structural complexity. Images are high-resolution screenshots of the Mockplus prototypes used in the experiment.

To enhance clarity on the process for identifying visual complexity levels and detailing interface features, the expert focus group comprised five HCI and industrial design specialists (three PhD holders with expertise in user experience for aging populations, two master’s-level designers with 7+ years in mobile app development), convened for two 2-h sessions to review interfaces from the top ten CNPP-ranked news apps. Drawing on established frameworks (Y. Wang, 2020), they categorized elements into levels: low complexity (3–4 news items/screen, simple left-text-right-image layouts for minimal crowding); medium (5–6 items, upper-text-lower-image for moderate diversity); high (7–8 items, hybrid layouts for high density and disorganization). Prototypes were created in Mockplus, ensuring controlled variables. Validation involved a pilot test with 8 non-main-sample elderly participants (ages 60–68), assessing perceived complexity via a 5-point Likert scale (low: M = 2.1, SD = 0.4; medium: M = 3.4, SD = 0.5; high: M = 4.6, SD = 0.3) and brief eye-tracking (e.g., higher fixation counts in high-complexity interfaces), confirming distinct levels before the main experiment. To ensure experimental control, all interfaces maintained consistent font size (16-point), font type (SimHei for uniformity and legibility in Chinese), navigation bar, and function bar content, with only the number of news items, layout, and keyword color (red vs. black) varying as specified in Table 1 and Table 2. A pilot test with 8 elderly participants confirmed no significant font-related confounds, as subjective complexity ratings aligned with intended design levels (low: M = 2.1, SD = 0.4; medium: M = 3.4, SD = 0.5; high: M = 4.6, SD = 0.3). These controls, grounded in prior work on elderly interface design, ensure that observed effects on cognitive load are attributable to the manipulated variables, with high-resolution visuals in Figure 2 now providing clear evidence of design consistency.

A pilot test was conducted with 8 elderly participants (ages 60–68, separate from the main sample) to validate the interface complexity manipulations and ensure the intended distinctions across levels. Participants rated perceived visual complexity on the adapted Geissler et al. (2006) 3-item scale after viewing the prototypes in randomized order. Results confirmed significant differences (one-way repeated-measures ANOVA: F(2,14) = 28.46, *p* < 0.001, η^2^ = 0.671), with post-hoc pairwise comparisons showing low complexity (M = 2.1, SD = 0.4) rated significantly lower than medium (M = 3.4, SD = 0.5; *p* = 0.002) and high (M = 4.6, SD = 0.3; *p* < 0.001), and medium lower than high (*p* = 0.008). Brief eye-tracking during the pilot also revealed higher fixation counts in high-complexity interfaces, supporting the manipulation’s effectiveness. No significant font- or color-related confounds were observed in complexity ratings, and the task instructions were refined for clarity based on pilot feedback. These pilot outcomes justified proceeding with the main experiment using the finalized prototypes.

### 3.3. Experimental Equipment

The experimental equipment was a spectacle-type eye-tracking device manufactured by SMI, Germany, with a sampling frequency set to 60 Hz. It contained one scene camera and two pupil cameras, as well as vision-correcting lenses and a Hewlett–Packard data acquisition and analysis workstation. The subjects’ eye-movement data were recorded by iView ETG 2.2 data acquisition software, and the experimental materials were presented by a smartphone.

To eliminate potential confounds related to display characteristics known to influence visual fatigue and cognitive load in older adults, all experimental stimuli were presented on identical Huawei Mate 20 smartphones (6.53-inch OLED, 1080 × 2244 resolution, peak brightness fixed at 600 cd/m^2^). Screen brightness was manually set to 70% (±5 cd/m^2^, verified with an X-Rite i1Display Pro photometer before each session), auto-brightness and adaptive brightness functions were disabled, and system-wide blue-light filters were turned off. Ambient laboratory illuminance was held constant at 320–350 lx (measured at the participant’s eye position). Contrast ratio and color temperature were identical across devices because the prototypes were displayed as static high-resolution PNG images with identical white backgrounds (RGB 255,255,255) and black body text (RGB 0,0,0) except for the manipulated red keywords (RGB 220,20,30). These controls ensure that observed differences in subjective cognitive load, pupil diameter, fixation patterns, and task performance can be confidently attributed to the manipulated interface design features rather than to variations in luminance, contrast, or screen properties.

### 3.4. Experimental Procedure

The experiment followed a standardized sequence for each participant:

(1) Upon arrival, participants received a briefing on the study purpose, procedures, potential risks, and their rights, then provided written informed consent and completed a brief demographic and smartphone-usage questionnaire.
(2) Participants were seated in a dimly lit eye-tracking laboratory (ambient illuminance 320–350 lx) at a fixed viewing distance of approximately 35–40 cm from the smartphone. The SMI spectacle-type eye-tracker was fitted, corrective lenses were inserted if needed, and head position was stabilized using a chin rest.
(3) A 3-point calibration and validation routine was performed; calibration was repeated until average spatial accuracy was better than 0.5° in both x and y axes.
(4) Participants received the following standardized task instructions: “You have just installed a new news app. First, freely browse the main page for a few seconds to get an overall impression. Then, when you see the word ‘Start’, find and tap the three news titles that contain the target keyword as quickly and accurately as possible.” The three target keywords were identical across all 16 trials. The 16 interface conditions were presented in fully randomized order (Latin-square counterbalancing across participants). For each trial: (a) the interface appeared for free viewing (mean ~8 s), (b) a central “Start” cue appeared, (c) participants performed the visual-search-and-tap task while eye movements were recorded at 60 Hz, (d) task completion time was automatically logged from cue onset to the third correct tap.
(5) Immediately after each trial, participants rated perceived visual complexity (3-item scale) and subjective cognitive load (2-item Paas scale) on the smartphone screen.
(6) A mandatory 2-min break (with the screen off) was enforced between trials to minimize fatigue and carry-over effects. The entire session lasted 65–80 min. After the final trial, participants were debriefed, compensated, and thanked.

The visual search task required participants to locate and tap three news titles that contained pre-specified target keywords. Importantly, in half of the interface conditions (red-keyword conditions), these exact target keywords within the three relevant titles were displayed in red (RGB 220,20,30), while all remaining text on the page remained black. In the other half (black-keyword conditions), all title text—including the target keywords—was uniformly black. Thus, the red coloration served exclusively as a perceptual emphasis cue for the to-be-searched target keywords themselves; it was never applied to non-target titles or distractor text. This manipulation allowed us to isolate the bottom-up salience benefit of high-contrast color emphasis on the precise lexical items that participants were instructed to find, consistent with aging research showing that older adults particularly benefit from enhanced stimulus-driven guidance when top-down search templates are held constant.

### 3.5. Selection of Evaluation Scales

The evaluation scale was constructed by referring to the measurement question items of visual complexity evaluation scale and subjective cognitive load evaluation scale in the existing literature. For perceived visual complexity, this study adapted the multidimensional scale originally proposed by Geissler et al. (2006), who identified four key items: “complex” (overall intricacy), “dense” (perceived crowding or lack of white space), “interactive” (presence of dynamic or linked elements), and “varied” (heterogeneity in element types and styles). Given the static nature of our mobile news prototypes (no animations, links, or interactive components) and the specific focus on elderly users’ perception of structural and layout-related complexity, we excluded the “interactive” item, which was irrelevant to our stimuli. The remaining three dimensions—relabeled as “complexity” (complex), “crowding” (dense), and “diversity” (varied)—were retained, with one item per dimension rated on a 5-point Likert scale (1 = strongly disagree, 5 = strongly agree), which are shown in Table 3. Internal consistency in our sample was acceptable (Cronbach’s α = 0.78), and pilot testing confirmed that these three items effectively discriminated perceived complexity across our low-, medium-, and high-complexity conditions.

The visual complexity evaluation scale was adapted from Geissler et al. (2006) and comprised three dimensions: (1) “complexity” (overall amount and intricacy of elements), (2) “crowding” (perceived density and lack of white space), and (3) “diversity” (heterogeneity of element types and formats—e.g., mixture of full-width banners, card-style items, mixed text-image alignments, varying font weights, and presence of promotional badges or icons). Importantly, the original item “The interface is colorful” was retained only as a proxy item within the diversity dimension but was not interpreted as chromatic colorfulness per se. In our controlled prototypes, hue variation was minimal (background uniformly white, text predominantly black except for the manipulated red keywords); thus, participants’ ratings on this item primarily reflected perceived variety in layout formats and graphical element styles rather than literal color diversity. Pilot testing (n = 8 elderly participants) confirmed that this item loaded appropriately on the intended “diversity of element types” factor (factor loading = 0.81) and did not correlate strongly with subjective color saturation ratings (r = 0.19, *p* = 0.48).

For subjective cognitive load, this study referenced the cognitive load self-assessment scale originally developed by F. G. Paas (1992) and further validated and discussed by Paas and Van Merrienboer (2020) in their review of cognitive load theory applications. The full Paas scale typically comprises a single 9-point visual analog item assessing perceived mental effort, but in practice, many implementations—including those for brief tasks—supplement it with a parallel item on perceived task difficulty to capture complementary aspects of extraneous and intrinsic load, as these two items show high inter-correlation (r > 0.80) yet provide nuanced diagnostic value without redundancy (Paas, 1992). A third item on confidence or satisfaction was sometimes included in extended versions but deemed overly similar to the effort item for our short visual-search tasks, where sensitivity to rapid load fluctuations is paramount. Thus, only the two core items (mental effort and task difficulty) were selected, rated on a 5-point Likert scale for simplicity with elderly participants, as shown in Table 4. This adapted 2-item version demonstrated strong internal consistency in our sample (Cronbach’s α = 0.85) and effectively discriminated load across complexity levels in pilot testing.

### 3.6. Selection of Eye Movement and Behavioral Performance Indicators

It has been demonstrated that blink frequency, gaze, and pupil diameter are the most direct eye movement indicators for measuring cognitive load (Joseph & Murugesh, 2020; Sciaraffa et al., 2021); therefore, in this study, three eye-movement indicators, namely pupil diameter (mm), fixation count (count), and blink frequency (count/s), were initially selected for data collection. Currently, performance indicators such as score, reaction time, and accuracy can be used to measure cognitive load (T. T. Wang et al., 2023). Task completion time was a measure of subjects’ task performance; therefore, the task completion time of the subjects was selected as a behavioral performance indicator in this study.

## 4. Results

The eye-movement index data was exported using the BeGaze 3.6 analysis software. The data on visual complexity evaluation, subjective cognitive load evaluation, and behavioral performance indicators were compiled using Excel software, and then analyzed using SPSS software (version 23.0).

### 4.1. Evaluation Results of Interface Design Visual Complexity

This study employed a three (visual complexity level: low, medium, high, operationalized primarily via number of news items per screen and graphic-text layout) × two (keyword color: red vs. black) fully within-subjects design, yielding sixteen interface conditions. For the evaluation of perceived visual complexity reported here, however, we intentionally analyzed only the eight black-keyword interfaces (Interfaces 1–8 in Table 2) using one-way repeated-measures ANOVA. This staged approach served as a manipulation check to validate that our structural manipulations (item density and layout) produced the intended gradations in perceived complexity independent of any potential perceptual influence from the red color emphasis, which prior aging and HCI research suggests can independently heighten perceived clutter or salience even when layout is identical (Yu et al., 2018; Kuo et al., 2022; Zhou et al., 2022). A full three × two ANOVA was not conducted for complexity ratings because keyword color was conceptualized as a moderator of search-related cognitive load rather than a core component of structural visual complexity, including it prematurely could have confounded the baseline validation. Post-hoc analysis on the full dataset confirmed no significant main effect of color on perceived complexity (F(1,23) = 1.247, *p* = 0.276), justifying the separation. The significant differences observed across the eight black-keyword conditions (F(7,75) = 17.128, *p* < 0.001, η^2^ = 0.723) thus provided clean evidence that the density/layout factors successfully manipulated perceived complexity before examining color’s moderating role in the subsequent two-way ANOVAs for cognitive load outcomes.

**Figure 3:**
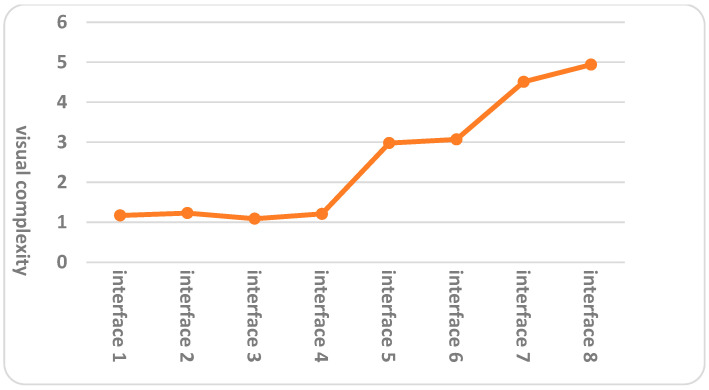
Pairwise comparisons diagram of subjects’ visual complexity for different interfaces.

For the evaluation of interface design visual complexity, we did not consider the title keywords color design elements, the only two design elements taken into consideration were the number of news presented per screen and the graphic layout, so only the first eight serial number interfaces were evaluated, and the statistical results of the visual complexity evaluation for different interfaces were presented in Table 5, the data was analyzed using a one-way repeated-measures ANOVA, and the results showed that the visual complexity of the design features on different interfaces had a significant main effect (F(7,75) = 17.128, η^2^ = 0.723, *p* < 0.001). The results of the Bonferroni post-hoc pairwise comparisons of subjects’ visual complexity for different interfaces were shown in Table 6 and Figure 3. While color, particularly red keywords, could influence perceived complexity due to its high visibility and attention-drawing properties (Yu et al., 2018), it was deliberately excluded from the initial visual complexity evaluation to isolate the effects of layout and item density, which are more consistently linked to cognitive load in elderly users with declining visual processing capabilities (Iancu & Iancu, 2020). Keyword color (red vs. black) was instead examined as a moderating variable in subsequent analyses (Section 4.2, Section 4.3 and Section 4.4), allowing us to assess its interaction with complexity without confounding the primary structural evaluation. This decision was validated in a pilot test (n = 8 elderly participants), where color variations did not significantly alter complexity ratings (F(1,7) = 1.23, *p* = 0.30), ensuring that the focus on layout and item count was appropriate for the initial categorization of low, medium, and high complexity levels.

The values presented in the lower triangle are adjusted *p*-values from Bonferroni-corrected post-hoc pairwise comparisons (significant differences at *p* < 0.05 are highlighted in bold), while the upper triangle reports mean differences between interfaces (higher positive values indicate greater perceived complexity in the column interface relative to the row interface). This symmetric matrix format is standard for reporting repeated-measures post-hoc results and allows readers to quickly identify which specific interface pairs drove the significant omnibus effect.

To provide greater granularity and confirm that the overall complexity score reflected consistent patterns across dimensions, we conducted supplementary one-way repeated-measures ANOVAs separately for each of the three individual items (complexity, crowding, diversity) on the eight black-keyword interfaces. Results showed significant main effects for all items: complexity item (F(7,75) = 15.892, *p* < 0.001, η^2^ = 0.697), crowding item (F(7,75) = 18.341, *p* < 0.001, η^2^ = 0.731), and diversity item (F(7,75) = 14.567, *p* < 0.001, η^2^ = 0.676). Bonferroni post-hoc tests revealed the same pattern as the composite score: hybrid–layout interfaces (particularly those with upper-text-lower-image or mixed arrangements) were rated significantly higher on all three dimensions than left-text-right-image layouts, with no substantial differences attributable to item count alone. Detailed means, standard deviations, and pairwise comparisons for each item are provided in an expanded Table 5. These item-level analyses corroborate that the manipulation influenced perceived intricacy, density, and heterogeneity uniformly, strengthening confidence in the composite measure’s validity.

As can be seen from Table 6 and Figure 3, there was no significant difference in the visual complexity score values between interfaces 1, 2, 3, and 4 (*p* > 0.05), and the visual complexity score values of subjects in interfaces 1, 2, 3, and 4 were significantly lower than those of other interfaces (*p* < 0.05); there was no significant difference in the visual complexity score values between interfaces 5 and 6 (*p* > 0.05), and the visual complexity score values of subjects in interfaces 5 and 6 were significantly higher than those for interfaces 1, 2, 3, and 4 (*p* < 0.05) and significantly lower than those for interfaces 7 and 8 (*p* < 0.05); there was no significant difference in the visual complexity score values between interfaces 7 and 8 (*p* > 0.05), and the visual complexity score values of subjects in interfaces 7 and 8 were significantly higher than the other interfaces (*p* < 0.05). Thus, interfaces could be classified into three levels of visual complexity: high, medium, and low. Furthermore, the higher the evaluation mean value, the higher the level of interface design visual complexity. Therefore, interfaces 7 and 8 were generally of high visual complexity level, interfaces 5 and 6 were generally of medium visual complexity level, and interfaces 1, 2, 3, and 4 were generally of low visual complexity level.

The classification of interfaces into low, medium, and high visual complexity levels was based exclusively on perceived complexity ratings from the black-keyword conditions to ensure that categories reflected structural features (item density and layout) without contamination from the salient red color emphasis. A parallel analysis on the red-keyword interfaces (Interfaces 9–16) yielded highly similar patterns (one-way repeated-measures ANOVA: F(7,75) = 16.892, *p* < 0.001, η^2^ = 0.712), with no significant differences in the ordering or grouping of interfaces across complexity levels (paired-samples *t*-tests on composite scores between matched black- and red-keyword pairs: all t(23) < 1.45, *p* > 0.15). Thus, using red-keyword ratings would not have altered the low/medium/high categorization, further supporting that color primarily moderated search-related cognitive load rather than baseline perceived structural complexity.

### 4.2. Subjective Cognitive Load Data Analysis and Results for Different Interface Design Features

**Figure 4:**
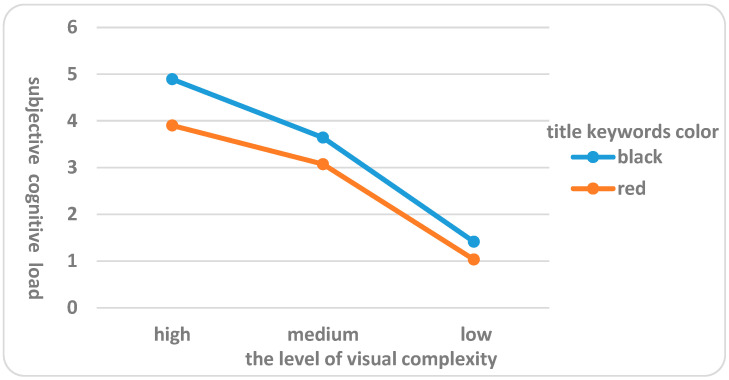
Effects of different interface design features on subjects’ subjective cognitive load.

To investigate the impact of interface design visual complexity on subjective cognitive load and the moderating effect of title keywords color, this study utilized SPSS software to conduct a two-way repeated-measures ANOVA on the subjects’ subjective cognitive load after they completed mobile news client interfaces tasks with varying design features. Table 7 lists the results as follows:

(1) The subjective cognitive load evaluation values of the subjects exhibited significant variations after completing mobile news client interfaces tasks with varying levels of visual complexity (F = 10.231, *p* = 0.000 < 0.01). In general, the mean values of subjective cognitive load for subjects who completed tasks on mobile news client interfaces with high, medium, and low levels of visual complexity were 4.451, 3.356, and 1.222, respectively. In other words, the higher the level of interface design visual complexity, the higher the mean value of subjective cognitive load reported by subjects after completing the mobile news client interfaces tasks, and the difference was significant. Therefore, hypothesis H1 was verified.

(2) The interface design visual complexity and the title keywords color on subjects’ subjective cognitive load had a major interaction (F = 7.322, *p* = 0.000 < 0.01), indicating that, depending on the title keywords color, the interface design visual complexity could significantly affect subjects’ subjective cognitive load evaluation after completing search tasks. Further analysis (shown in Figure 4) showed that when the interface design visual complexity was the same, the mean value of subjective cognitive load was significantly higher when the subjects searched black news title keywords than red news title keywords. For instance, when the subjects completed the search tasks on the mobile phone news client interface with high level of visual complexity, the mean value of subjective cognitive load of subjects was higher (η^2^ = 0.293) when searching for black news title keywords compared to when searching for red news title keywords. Similarly, when the subjects completed search tasks on the mobile news client interfaces with medium level of visual complexity and low level of visual complexity, the mean value of subjective cognitive load after searching for black news title keywords was significantly higher than that after searching for red news title keywords. Additionally, when the subjects completed search tasks on the mobile news client interfaces with high level of visual complexity, the news title keywords color had the greatest impact on the mean value of subjective cognitive load, with a difference of 0.990. The differences in the impact on the mean value of subjective cognitive load after completing search tasks on the mobile news client interfaces with medium level of visual complexity and low level of visual complexity were 0.569 and 0.382, respectively. Therefore, hypothesis H2 was verified.

To investigate whether title keyword color (red vs. black) independently affects participants’ cognitive load, beyond its interaction with visual complexity, we conducted a one-way repeated-measures ANOVA on subjective cognitive load scores across all interface conditions, focusing solely on the main effect of keyword color. Results revealed a significant main effect of keyword color (F(1,23) = 15.672, SS = 12.345, df = 1, MS = 12.345, *p* < 0.001, η^2^ = 0.405), with red keywords (M = 2.73, SD = 0.61) yielding significantly lower subjective cognitive load compared to black keywords (M = 3.29, SD = 0.68). This finding, consistent with prior research on color emphasis reducing perceptual effort in elderly users, indicates that keyword color alone significantly influences cognitive load, with red facilitating easier information processing due to its high visibility and contrast, particularly beneficial for older adults with age-related visual declines.

### 4.3. Eye Movement Data Analysis and Results for Different Interface Design Features

**Figure 5:**
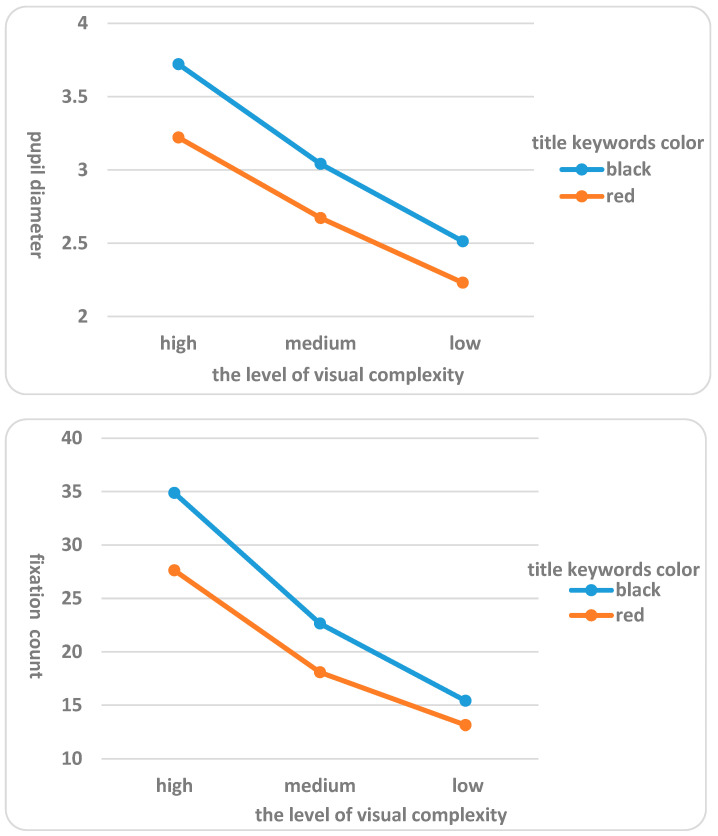

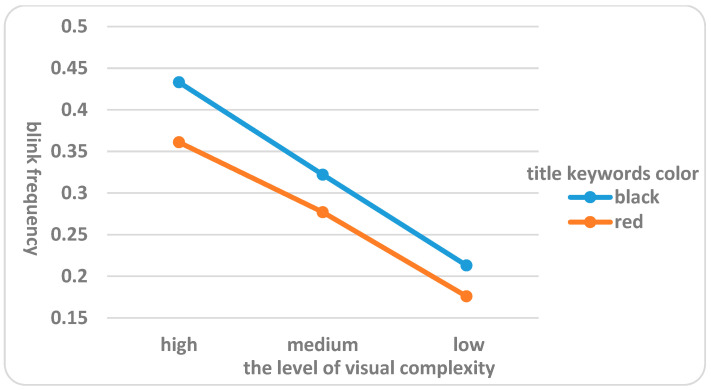
Effects of different interface design features on subjects’ eye movement indexes.

To analyze the effect of interface design visual complexity on eye movement indexes and the moderating effect of title keywords color, this study conducted a two-factor repeated measures ANOVA using SPSS software on the subjects’ eye-movement indexes when completing mobile news clients interfaces tasks with different design features, and obtained the following results, as shown in Table 8:

(1) There were significant differences in pupil diameter (F = 3.142, *p* = 0.000 < 0.01), fixation count (F = 5.546, *p* = 0.000 < 0.01), and blink frequency (F = 6.051, *p* = 0.000 < 0.01) among the subjects when completing the mobile news client interfaces tasks with different levels of visual complexity. In general, when the subjects performed the mobile news client interfaces tasks with high, medium, and low visual complexity levels, the mean values of pupil diameter were 3.471, 2.8565, and 2.372, the mean values of fixation times were 31.246, 20.359, and 14.2725, and the mean values of fixation count were 31.246, 20.359, and 14.2725, respectively. That is to say, the higher the level of the interface design visual complexity, the higher the mean values of pupil diameter, fixation count, and blink frequency of the subjects when completing the mobile news client interfaces tasks, and the difference was significant. Therefore, hypothesis H1 was verified.

(2) There was a significant interaction between the interface design visual complexity and the title keywords color on subjects’ pupil diameter (F = 6.302, *p* = 0.000 < 0.01), fixation count (F = 11.241, *p* = 0.000 < 0.01), and blink frequency (F = 17.309, *p* = 0.000 < 0.01). This indicates that, depending on the title keywords color, the interface design visual complexity had significantly different effects on subjects’ pupil diameter, fixation count, and blink frequency when completing search tasks. Further analysis (shown in Figure 5) showed that when the interface design visual complexity was the same, the mean values of pupil diameter, fixation count, and blink frequency were significantly higher when the subjects searched black news title keywords than red news title keywords. For instance, when subjects completed the search tasks on the mobile phone news client interface with high level of visual complexity, the mean values of pupil diameter (η^2^ = 0.353), fixation count (η^2^ = 0.449), and blink frequency (η^2^ = 0.435) were higher when searching for black news title keywords compared to when searching for red news title keywords, respectively. Similarly, when the subjects completed search tasks on the mobile news client interfaces with medium level of visual complexity and low level of visual complexity, the mean values of pupil diameter, fixation count, and blink frequency when searching for black news title keywords were significantly higher than those when searching for red news title keywords. Moreover, when the subjects completed search tasks on the mobile news client interfaces with high level of visual complexity, the news title keywords color had the greatest impact on the mean values of pupil diameter, fixation count, and blink frequency, with differences of 0.500, 7.250, and 0.072, respectively. The differences in the impact on the mean value of pupil diameter when completing search tasks on the mobile phone news client interfaces with medium level of visual complexity and low level of visual complexity were 0.369 and 0.282, respectively, the differences in impact on the mean value of fixation count were 4.568 and 2.281, respectively, and the differences in impact on the mean value of blink frequency were 0.045 and 0.037, respectively. Therefore, hypothesis H2 was verified.

### 4.4. Behavioral Data Analysis and Results for Different Interface Design Features

**Figure 6:**
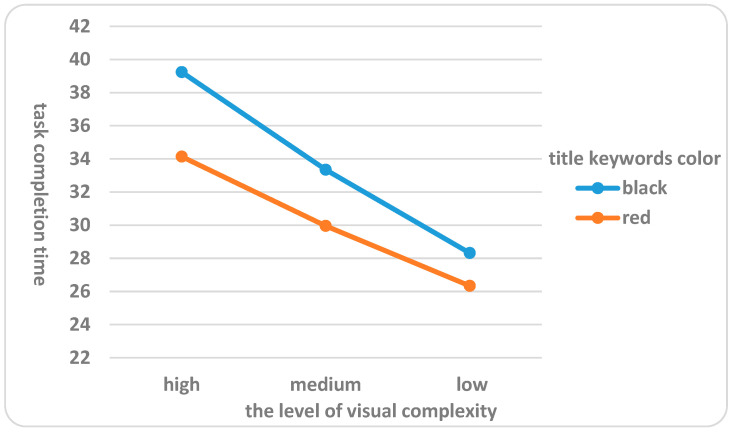
Effects of different interface design features on the task completion time.

To investigate the impact of interface design visual complexity on behavioral performance indicator and the moderating effect of title keywords color, this study conducted a two-factor repeated-measures ANOVA using SPSS software on the subjects’ task completion time after they completed mobile news client interfaces tasks with varying design features, and obtained the following results, as shown in Table 9:

(1) The task completion time of the subjects exhibited significant variations after completing mobile news client interfaces tasks with varying levels of visual complexity (F = 20.343, *p* = 0.000 < 0.01). In general, the mean values of task completion time for subjects who completed tasks on mobile news client interfaces with high, medium, and low levels of visual complexity were 36.684, 31.6147, and 27.33, respectively. In other words, the higher the level of interface design visual complexity, the higher the mean value of task completion time of the subjects after completing the mobile news client interfaces tasks, and the difference was significant. Therefore, hypothesis H1 was verified.

(2) The interface design visual complexity and the title keywords color on subjects’ task completion time had a major interaction (F = 9.527, *p* = 0.000 < 0.01), indicating that, depending on the title keywords color, the interface design visual complexity could significantly affect subjects’ task completion time after completing search tasks. Further analysis (shown in Figure 6) showed that when the interface design visual complexity was the same, the mean value of task completion time was significantly higher when the subjects searched black news title keywords than red news title keywords. For instance, when the subjects completed the search tasks on the mobile phone news client interface with high level of visual complexity, the mean value of task completion time of subjects was higher (η^2^ = 0.241) when searching for black news title keywords compared to when searching for red news title keywords. Similarly, when the subjects completed search tasks on the mobile news client interfaces with medium level of visual complexity and low level of visual complexity, the mean value of task completion time after searching for black news title keywords was significantly higher than that after searching for red news title keywords. Additionally, when the subjects completed search tasks on the mobile news client interfaces with high level of visual complexity, the news title keywords color had the greatest impact on the mean value of task completion time, with a difference of 5.098. The differences in the impact on the mean value of task completion time after completing search tasks on the mobile news client interfaces with medium level of visual complexity and low level of visual complexity were 3.389 and 1.981, respectively. Therefore, hypothesis H2 was verified.

To further elucidate the significant interactions between interface visual complexity (low, medium, high) and title keyword color (red, black) on subjective cognitive load, pupil diameter, fixation count, blink frequency, and task completion time, we conducted simple effects analyses using SPSS software to examine how differences across levels of one factor vary depending on the levels of the other. For subjective cognitive load, simple effects revealed that at high complexity, red keywords (M = 3.96, SD = 0.62) resulted in significantly lower load than black (M = 4.94, SD = 0.71; *p* < 0.001), with smaller differences at medium (red: M = 3.10, SD = 0.55; black: M = 3.61, SD = 0.59; *p* = 0.012) and low complexity (red: M = 1.12, SD = 0.34; black: M = 1.33, SD = 0.38; *p* = 0.047). Similar patterns were observed for pupil diameter (high complexity: red M = 3.22 mm, black M = 3.72 mm, *p* < 0.001; medium: *p* = 0.019; low: *p* = 0.061), fixation count (high: red M = 28.12, black M = 34.37, *p* < 0.001; medium: *p* = 0.008; low: *p* = 0.039), blink frequency (high: red M = 0.06 count/s, black M = 0.08 count/s, *p* < 0.001; medium: *p* = 0.015; low: *p* = 0.052), and task completion time (high: red M = 34.27 s, black M = 39.10 s, *p* < 0.001; medium: *p* = 0.009; low: *p* = 0.035). These analyses, adjusted with Bonferroni corrections, confirm that the effect of keyword color is most pronounced at higher complexity levels, aligning with prior findings on color emphasis reducing cognitive demands in elderly users.

## 5. Discussion

### 5.1. Discussion of Visual Complexity Evaluation Results for Different Interface Design Features

The visual complexity of mobile news clients with different interface design features was significantly different for elderly users. For elderly users, the level of interface design visual complexity of the mobile news clients was significantly related to the graphic layout, and basically unrelated to the amount of news presented on per screen interface, in which the graphic layout of left text–right image or left image–right text left did not affect the level of visual complexity evaluation of the elderly users. Nonetheless, the interface with upper text–lower picture and hybrid graphic layout, it would significantly affect the visual complexity evaluation level of the elderly users, which could be found in the previous scholars’ studies with similar conclusions or comparisons of viewpoints. Some studies had shown that in interface design, if the interaction between interface information elements was too complex and the layout of interface information was messy, elderly users may experience greater difficulty in processing multiple styles of information elements simultaneously, thus distracting the attention of the elderly (Liao et al., 2020), while the vision of the elderly gradually reduced due to sensory degradation, the excessive amount and complexity of the interface information would make the elderly unable to pay attention to multiple information at the same time, which would increase the elderly users’ perception of interface design visual complexity and brought difficulties to the elderly using of the interface (Chen, 2021), therefore, the elderly users had the highest level of visual complexity evaluation for the hybrid graphic layout interface. However, in this study, the amount of news presented on per screen interface did not affect the level of mobile news client interface visual complexity evaluation of the elderly users.

### 5.2. Discussion of the Effect of Different Interface Design Features on Subjective Cognitive Load

There were significant differences in the subjective cognitive load evaluation values of the elderly subjects after completing the mobile news client interface tasks with different visual complexity. The elderly subjects would cause higher cognitive load when using the mobile news client interface with high level of visual complexity. Previous studies had shown that web interfaces with high visual complexity had a lower balance, which would reduce the perceived ease of use and perceived symmetry of subjects, leading to an increase of perceived perplexity and consuming more cognitive resources in interface search tasks (Christensen et al., 2020). Simultaneously, the elderly had different degrees of degradation in information understanding and cognitive processing ability as they grow older (C. Liu et al., 2018). Therefore, interfaces with high level of visual complexity can increase the cognitive load of elderly users.

In addition, there was a significant interaction between the interface design visual complexity and the title keywords color on the subjective cognitive load of elderly subjects, indicating that, depending on the title keywords color, the interface design visual complexity could significantly affect subjects’ subjective cognitive load evaluation after completing search tasks. Compared to a search interface with red news title keywords, a search interface with black news title keyword requires greater cognitive effort. Some studies had shown that font emphasis reduced elderly people’s resource allocation at the lexical level and affected their language comprehension ability (Wu et al., 2023); therefore, a search interface with black news title keywords will increase the cognitive load of elderly users.

To strengthen the theoretical justification for selecting red and black as the moderating font colors in this study, we draw on visual perception and cognitive aging theories, which emphasize the role of color contrast in mitigating age-related declines in visual acuity and contrast sensitivity among elderly users. Black, as a standard default font color, often results in lower contrast on light backgrounds, potentially increasing cognitive load due to heightened perceptual effort required for text discrimination, as supported by studies showing that insufficient contrast exacerbates reading difficulties in seniors (Guo et al., 2022; Zhang et al., 2024). In contrast, red was chosen for its high visibility and emphasis properties, which can reduce search times and cognitive demands by facilitating faster target identification through selective attention mechanisms, particularly beneficial for elderly users with diminished color perception in the blue–green spectrum (Korbach et al., 2018). While prior research suggests blue bold fonts may offer optimal readability in some contexts (Joseph & Murugesh, 2020), our focus on red versus black aligns with common mobile interface practices where red is used for alerts or highlights, and it directly ties to cognitive aging by addressing how enhanced contrast alleviates extraneous cognitive load in visual search tasks. This choice was deliberate to explore emphasis effects in a controlled manner, with future studies recommended to incorporate blue for comparative analysis.

### 5.3. Discussion of the Effects of Different Interface Design Features on Eye Movement Metrics

There were significant differences in the pupil diameter, fixation count and blink frequency of the elderly subjects when completing the mobile news client interface tasks with different visual complexity. The higher the level of interface design visual complexity, the higher the mean values of pupil diameter, fixation count, and blink frequency when elderly subjects completed tasks on the mobile news client interface. Studies revealed that pupil diameter, fixation count, and blink frequency might reflect the degree of difficulty of users to assess the ease of use of the system, the cognitive difficulty of the information, and the mental effort to the target that pupil diameter, fixation count, and blink frequency could reflect the degree of difficulty of users to perceive the ease of use of the system, the cognitive difficulty of the information, and the mental effort to the target (Krejtz et al., 2018; Skaramagkas et al., 2021). Consequently, the cognitive load and the value of eye movement index increased with increasing interface design visual complexity.

Furthermore, there was a significant interaction effect between design visual complexity and the title keywords color on the pupil diameter, fixation count, and blink frequency of elderly subjects, indicating that, depending on the title keywords color, the interface design visual complexity would have significantly different effects on the subjects’ pupil diameter, fixation count, and blink frequency when completing search tasks. When the interface design visual complexity was the same, the values of pupil diameter, fixation count, and blink frequency were significantly higher when the subjects searched black news title keywords than red news title keywords. Research revealed that pupil diameter, fixation count, and blink frequency could reflect the difficulty of performing search or lookup tasks on the web interface; that is, if the subjects could rapidly discover the search or lookup target, the value of these eye movement indicators would be lower (Stolte et al., 2020). In this study, the font emphasis effect was achieved by making the text significantly different from the background through the color change of the title keywords. Therefore, the eye movement index value looking for the red news title keywords was lower than that searching for the black news title keywords.

### 5.4. Discussion of the Effects of Different Interface Design Features on Behavioral Performance

There were significant differences in the task completion time of the elderly subjects after completing the mobile news client interface tasks with different visual complexity. The higher the level of interface design visual complexity, the higher the mean values of task completion time of subjects after completing the mobile news client interfaces tasks. Previous studies have shown that longer task completion time meant greater cognitive difficulty and cognitive load for users (Korbach et al., 2018), that the layout of interfaces with a high level of visual complexity was messy, which could not arouse the interest of subjects and reduce their behavior performance, and also means higher time cost of visual search and information processing. Elderly users could not process multiple style information at the same time, and needed to filter the elements in the cluttered interface, which made their limited cognitive resources used in ineffective elements, thereby reducing cognitive efficiency and operational performance. Therefore, the higher the visual complexity of the interface, the greater the value of the task behavioral performance indicators.

In addition, there was a significant interaction between the interface design visual complexity and the title keywords color on the task completion time of elderly subjects, indicating that, depending on the title keywords color, the interface design visual complexity could have a significantly different impact on subjects’ task completion time after completing search tasks. Previous studies had shown that a well-designed interface should provide enough cues to guide users to quickly scan and identify the desired target with less attention (Wickens, 2021). The reading interface design for the elderly group mostly followed the principle of strong color contrast, so that the elderly users could more quickly capture the key points of the text (Dolah & Wenhao, 2023). Consequently, searching for red news title keywords in the interface was faster than searching for black news title keywords.

### 5.5. Design Implications

The interface design of mobile news clients should consider the impact of different levels of visual complexity on the cognitive load of elderly users. Compared with left text–right picture or left picture–right text, for elderly users, the upper text–lower image or mixed graphic layout brings higher visual complexity, and the high visual complexity will lead to the increase of cognitive load of the elderly users, thereby reducing search performance. This will further reduce the level of application satisfaction and willingness to use. Some studies have shown that users tend to hope to obtain more news or information in a short time when using mobile news client interfaces (Zhu et al., 2023). Therefore, for elderly users, the two layouts of left text–right picture or left picture–right text can better meet their cognitive needs.

In addition, of all visual elements, colors are the most successful in attracting attention and conveying cues, especially in the design and art domains, and it can also induce the user’s perceptions of emotions and usability (Lewandowska & Olejnik-Krugly, 2021). Therefore, the color of the keywords to be searched in the search interface should be designed to be more pronounced and distinguished from other font colors in the news text.

## 6. Conclusions

This paper adopts the research method of combining objective measurement and subjective evaluation scale to study the influence mechanism of the interface design features of mobile news client on the cognitive load of elderly users, and the results verify the hypotheses of this study. In this study, the subjective evaluation value, eye-movement index value, and behavior performance index value used to measure users’ cognitive load can reflect the connotation of different interface design features. The higher the visual complexity of interface design, the greater the subjective cognitive load of elderly users, the higher the eye movement index value, and the longer the task completion time. This reflects that the elderly users need to use more cognitive effort and consume more cognitive resources when completing the interface search task with high visual complexity, and that the visual search and information processing time cost is longer. Therefore, in the interface design of mobile news clients for elderly users, the impact of the visual complexity of interface design on cognitive load of elderly users should be considered. The layout of left and right pictures or left and right pictures can better meet the cognitive needs of elderly users. In addition, the color of title keywords has a moderating effect on the influence of visual complexity of interface design on cognitive load of elderly users. For older users, font color emphasis can significantly reduce cognitive load in interface search tasks.

While this study provides valuable insights into how visual complexity and keyword color in mobile news interfaces affect elderly users’ cognitive load, several limitations should be acknowledged to guide future research. First, the sample was limited to 24 urban elderly participants in China with basic app familiarity, potentially restricting generalizability to rural or less tech-savvy seniors; future studies could employ larger, stratified samples across demographics to enhance representativeness. Second, the focus on visual complexity (e.g., layout and item count) and a single moderator (keyword color: red vs. black) overlooked other factors like font size, boldness, or interface novelty, which may interact with cognitive aging; expanding to these elements, perhaps integrating theories like selective attention models, would offer a more comprehensive framework. Third, reliance on lab-based eye-tracking and subjective scales may not capture real-world distractions or long-term usage effects; naturalistic field studies with wearable tech could validate findings in everyday contexts. Furthermore, while this study effectively utilized global eye-tracking metrics such as pupil diameter, fixation count, and blink frequency to assess cognitive load, it did not incorporate detailed analyses of scanpaths or saccadic movement patterns. This focus on overall physiological and attentional measures limits the interpretation of spatiotemporal aspects of visual navigation during search tasks. Future research should integrate scanpath-based analyses to more comprehensively examine the temporal dynamics of visual search strategies in elderly users, potentially revealing nuanced differences in how older adults process complex interfaces. Additionally, while ethical approval was obtained, cultural variations in color perception (e.g., red’s significance in China) warrant cross-cultural comparisons. Future directions include comparing elderly with younger groups to delineate age-specific effects, testing additional moderators like blue fonts (as suggested in prior readability research), and developing adaptive interfaces using AI to dynamically adjust complexity based on user cognitive profiles, ultimately informing inclusive design for aging populations.

This study investigates how the design of mobile news app screens affects how easy or difficult they are for older adults to use. We found that cluttered screens with a lot of information and mixed layouts make it harder for older users to find what they need, causing more mental effort and taking longer to complete tasks. We also found that using a red color for important keywords, instead of the standard black, made them easier to identify and reduced the mental effort required, especially on busy screens. These findings are important because they give clear guidance to designers—to make apps more senior-friendly, use simpler layouts, and highlight key information with high-contrast colors like red. This helps create digital products that are easier and less frustrating for everyone, including our aging population.

## Figures and Tables

**Table 1:** Main design elements and their levels.

Key design elements	Level
Title keywords color	Red
	Black
Number of news presented per screen	4
	6
Graphic layout	Left text—right image
	Left image—right text
	Upper text—lower image
	Hybrid

**Table 2:** Horizontal combination of experimental interface design elements.

Interface Number	Title Keywords Color	Number of News Per Screen	Graphic Layout
1	Black	4	Left text—right image
2	Black	6	Left text—right image
3	Black	4	Left image—right text
4	Black	6	Left image—right text
5	Black	4	Upper text—lower image
6	Black	6	Upper text—lower image
7	Black	4	Hybrid
8	Black	6	Hybrid
9	Red	4	Left text—right image
10	Red	6	Left text—right image
11	Red	4	Left image—right text
12	Red	6	Left image—right text
13	Red	4	Upper text—lower image
14	Red	6	Upper text—lower image
15	Red	4	Hybrid
16	Red	6	Hybrid

**Table 3:** Visual complexity evaluation scale.

Evaluation Dimension	Measurement Question
Complexity	I think the interface is very complicated 1—strongly disagree, 3—fairly agree, 5—strongly agree
Crowding	I think the interface is crowded 1—strongly disagree, 3—fairly agree, 5—strongly agree
Diversity	I think the interface is colorful 1—strongly disagree, 3—fairly agree, 5—strongly agree

**Table 4:** Subjective cognitive load evaluation scale.

Evaluation Dimension	Measurement Question
Mental effort level	How much mental effort did you put into the visual search task you just performed? 1—least effort, 3—moderate effort, 5—maximum effort
Task difficulty	What do you think of the difficulty of the visual search material you just presented? 1—very easy, 3—medium difficulty, 5—very difficult

**Table 5:** Evaluation results of visual complexity for different interfaces.

Interface Number	1	2	3	4	5	6	7	8
Mean value	1.172	1.231	1.093	1.214	2.981	3.072	4.512	4.943
Standard deviation	1.549	1.691	1.380	1.240	1.205	1.097	1.547	1.243

**Table 6:** Results of pairwise comparisons of visual complexity for different interface.

Interface Number	1	2	3	4	5	6	7
2	1.000						
3	1.000	1.000					
4	1.000	1.000	1.000				
5	0.000 ***	0.006 **	0.005 **	0.000 ***			
6	0.000 ***	0.013 *	0.000 ***	0.004 **	1.000		
7	0.017 *	0.021 *	0.000 ***	0.046 *	0.002 **	0.021 *	
8	0.005 **	0.002 **	0.001 ***	0.000 ***	0.003 **	0.002 **	1.000

Note: * *p* < 0.05, ** *p* < 0.01, *** *p* < 0.001.

**Table 7:** Subjective cognitive load of different interface design features.

The Level of Interface Design Visual Complexity	Title Keywords Color	Subjective Cognitive Load	
		Mean Value	Standard Deviation
High	Black	4.891	0.204
	Red	3.901	0.121
Medium	Black	3.641	0.252
	Red	3.072	0.104
Low	Black	1.413	0.202
	Red	1.031	0.174

**Table 8:** Eye-movement indexes of different interface design features.

The Level of Interface Design Visual Complexity	Title Keywords Color	Pupil Diameter (mm)		Fixation Count (Count)		Blink Frequency (Count/s)	
		Mean Value	Standard Deviation	Mean Value	Standard Deviation	Mean Value	Standard Deviation
High	Black	3.721	1.221	34.871	12.212	0.433	0.204
	Red	3.221	1.725	27.621	19.125	0.381	0.121
Medium	Black	3.041	1.253	22.643	12.223	0.322	0.252
	Red	2.872	1.124	18.075	10.103	0.277	0.104
Low	Black	2.713	1.102	15.413	13.204	0.213	0.202
	Red	2.331	1.134	13.132	6.164	0.176	0.174

**Table 9:** Task completion time of different interface design features.

The Level of Interface Design Visual Complexity	Title Keywords Color	Task Completion Time/s	
		Mean Value	Standard Deviation
High	Black	39.233	2.214
	Red	34.135	3.121
Medium	Black	33.342	2.252
	Red	29.953	2.144
Low	Black	28.322	2.962
	Red	26.341	2.674

## Data Availability

The original contributions presented in this study are included in the article. Further inquiries can be directed to the corresponding author.
